# Food Safety in the Age of Next Generation Sequencing, Bioinformatics, and Open Data Access

**DOI:** 10.3389/fmicb.2017.00909

**Published:** 2017-05-23

**Authors:** Eduardo N. Taboada, Morag R. Graham, João A. Carriço, Gary Van Domselaar

**Affiliations:** ^1^National Microbiology Laboratory, Public Health Agency of Canada, WinnipegMB, Canada; ^2^Department of Biological Sciences, University of Lethbridge, LethbridgeAB, Canada; ^3^Department of Medical Microbiology and Infectious Diseases, Max Rady College of Medicine, University of Manitoba, WinnipegMB, Canada; ^4^Instituto de Microbiologia, Instituto de Medicina Molecular, Faculdade de Medicina, Universidade de LisboaLisbon, Portugal

**Keywords:** food safety, next-generation sequencing, genomic epidemiology, molecular typing, open data access

## Abstract

Public health labs and food regulatory agencies globally are embracing whole genome sequencing (WGS) as a revolutionary new method that is positioned to replace numerous existing diagnostic and microbial typing technologies with a single new target: the microbial draft genome. The ability to cheaply generate large amounts of microbial genome sequence data, combined with emerging policies of food regulatory and public health institutions making their microbial sequences increasingly available and public, has served to open up the field to the general scientific community. This open data access policy shift has resulted in a proliferation of data being deposited into sequence repositories and of novel bioinformatics software designed to analyze these vast datasets. There also has been a more recent drive for improved data sharing to achieve more effective global surveillance, public health and food safety. Such developments have heightened the need for enhanced analytical systems in order to process and interpret this new type of data in a timely fashion. In this review we outline the emergence of genomics, bioinformatics and open data in the context of food safety. We also survey major efforts to translate genomics and bioinformatics technologies out of the research lab and into routine use in modern food safety labs. We conclude by discussing the challenges and opportunities that remain, including those expected to play a major role in the future of food safety science.

## Introduction

The first complete sequence of a bacterial organism—*Haemophilus influenzae*—was generated in 1995, revealing for the first time the entire set of genetic information used to encode a free-living organism. Beyond the intrinsic scientific value of the 1.8 million base pair genome sequence and the nearly 1700 coding and non-coding genes within, this landmark scientific achievement is notable as the first demonstration that random shotgun sequencing combined with sophisticated computational methods can be used to successfully assemble a genome. The *H. influenzae* sequencing project also is notable for making the genome sequence data and the bioinformatics software used to assemble it freely available to the scientific community. Such sharing aimed to be (and was) consistent with the policies initially set out by the ongoing Human Genome Project (NIH-DOE, 2012), later codified in the 1996 Bermuda Principles (Marshall, 2001). The policies of open sharing of genomic data and open source release of bioinformatics software tools set out by these seminal sequencing efforts were instrumental in cementing openness into the scientific culture (Lord et al., 2005). The impact of such open policies for scientific and medical advancement have been profound, as the publicly available genomic data and bioinformatics tools used to analyze these data are now routinely applied in nearly every aspect of biological and medical research, including the field of food safety science.

The fields of genomics and bioinformatics have been invaluable for advancing food safety science, although their application until recently has been limited toward research and development of molecular diagnostic technologies. For example, genomics and bioinformatics have been crucial in developing the standard molecular typing technologies currently in routine use for laboratory-based identification and tracking of foodborne disease outbreaks - namely Pulse-Field Gel Electrophoresis (PFGE), Multi-Locus Sequence Typing (MLST), and Multi-Locus Variable-Number of Tandem Repeats Analysis (MLVA). These tests require substantial bioinformatics and genomics to develop, but require only modest bioinformatics and genomics to carry out. What little bioinformatics and genomics that are required to conduct these tests historically have been incorporated within the various standardized lab procedures and software systems, effectively hidden “out of sight” (even “out of mind”) to most end users. This situation would radically change with the introduction of new, massively high throughput sequencing technologies, commonly referred to as Next-Generation Sequencing (NGS).

Next-generation sequencing was first made commercially available in late 2005 with the introduction of the GS20 sequencer manufactured by 454 Life Sciences. This new technology combined microfabrication advancements with an innovative new sequencing methodology to cheaply and rapidly generate massive amounts of nucleic acid sequencing data. Over the next decade, two main NGS technologies emerged, primarily distinguished by the sequence fragment (“read”) length generated. Short read technologies, such as those incorporated into the platform lines currently manufactured by Illumina and Life Technologies, generate read lengths from ∼100 to ∼600 bp with low per-base error rates (typically less than 1%) (Goodwin et al., 2016). These technologies are routinely used to assemble draft genome sequences containing multiple contiguous segments (contigs) with high accuracy and good coverage (>95% for an average bacterial genome). Two distinct longer read technologies are incorporated into the Pacific Biosystems (PacBio; Pacific Biosciences of California, Inc.) and Oxford Nanopore (Oxford Nanopore Technologies Ltd.) line of sequencers. The latter technologies both exploit single molecule sequencing to produce read lengths ranging from 1,000 to nearly 100,000 bp, although they still suffer from relatively high error rates (15–30%) (Goodwin et al., 2016). A current strength of long read technologies lies in their contribution to generating “scaffolds” used for inter-connecting high quality contigs generated by short read technologies; in combination they permit efficient reconstruction of the draft genome. It is even possible (albeit expensive) to generate a high quality, “complete” bacterial draft genome using only long read technologies. Long read sequencing technologies have additional niche applications that may prove useful for future food science applications, as will be discussed below.

Perhaps the most important feature of NGS technologies is their ability to cheaply and quickly generate draft whole genome sequencing (WGS) data. This is especially true for microbial genomes, due to their smaller, more compact genomes relative to eukaryote genomes. The ability to routinely generate microbial draft genome sequence data has important applications in food safety science, particularly for foodborne disease surveillance and outbreak investigation. The conventional molecular typing technologies expose a mere fraction of the entire information contained within a foodborne pathogen’s genome, and thus provide limited ability to discriminate outbreak-related pathogen strains from unrelated, sporadically circulating strains. In contrast, WGS can theoretically reveal the entirety of the genome for a given microbial pathogen thereby allowing for the discrimination of strains that differ by a single nucleotide (amongst the millions of nucleotides comprising a typical bacterial pathogen genome). Although early pioneering studies applying WGS to outbreak analysis demonstrated much promise for this new technology, widespread recognition of its power would first occur in 2011.

## Genomics and Bioinformatics in the Limelight

The ability of NGS technology to resolve the source of an outbreak was famously demonstrated during the 2010 Haiti cholera outbreak, the worst cholera epidemic in recent history killing at least 10,000 people and sickening well over 600,000 (Centers for Disease Control and Prevention [CDC], 2014). At the time of the outbreak, two hypotheses predominated as to its origin. One hypothesis argued that an endogenous pathogenic strain had been introduced from coastal waters; the other hypothesis suggested the cholera was introduced by UN peacekeepers deployed to Haiti after training in Kathmandu during a reported cholera outbreak spanning the country of Nepal (Maharjan, 2010). Conventional PFGE-based typing was insufficient to discriminate the outbreak strain from other environmental strains, and from other cholera outbreak strains originating mainly in Africa and Southeast Asia. The United States Centers for Disease Control and Prevention (CDC) performed NGS on a handful of strains from the Haitian outbreak and immediately released the data to the public. The free and open availability of this data allowed global researchers to compare the genome sequences from the Haitian strains with genome sequences from their own *Vibrio cholerae* collections, which they also rapidly released into the public domain (Chin et al., 2011; Hendriksen et al., 2011; Reimer et al., 2011). None of these early genomic epidemiological investigations were by themselves sufficient to definitively trace the origin of the Haiti outbreak to the prior outbreak in Nepal; yet their combined genomic data, together with the available epidemiological data from the Haiti outbreak, provided overwhelming support to the “introduced outbreak strain” hypothesis that the outbreak was imported to Haiti from Nepal (Eppinger et al., 2014).

Shortly thereafter, a second major outbreak occurred that would have important consequences for food safety science: the 2011 Germany *Escherichia coli* O104:H4 outbreak. This large-scale outbreak of a novel strain of *E. coli* claimed over 50 lives and clinically affected a further 4,000 individuals (Grad et al., 2012). Following the example set by the Haiti cholera outbreak investigation teams, genome sequences for the O104 outbreak strains were immediately released to the public. The timing of the release coincided with the *Applied Bioinformatics for Public Health Microbiology* conference hosted at the Wellcome Trust Sanger Institute in Hinxton, United Kingdom, in the spring of 2011. The conference had assembled many of the world’s top bioinformatics scientists with expertise in microbial genomics, including the German researchers currently involved in the ongoing *E. coli* O104:H4 outbreak investigation. Using social media and other internet technologies, conference attendees joined with other researchers across the globe to perform the first crowdsourced, real-time analyses of the outbreak sequence data (Rasko et al., 2011). The *ad hoc* research group generated the outbreak pathogen’s draft genome sequence in under a day, and within a week they had designed molecular targets to distinguish the novel O104 outbreak strain from other circulating strains. Within that same short timeframe, they also determined the pathogen’s evolutionary origin and assessed its pathogenic potential (Boxrud et al., 2010; Chewapreecha et al., 2014). The extraordinary speed in which the novel O104 genome was characterized, largely as a result of the rapid public release of the pathogen genomic sequence data and its crowdsourced analysis, was widely reported in the scientific community (Mellmann et al., 2011; Owens, 2011; Rohde et al., 2011; Society for General Microbiology, 2011).

Beyond generating international headlines, these events, along with several other timely landmark genomic epidemiology investigations (Beres et al., 2010; Gilmour et al., 2010; Harris et al., 2010; Lewis et al., 2010; Gardy et al., 2011; Mutreja et al., 2011) spurred a grass-roots modernization movement. In the fall of 2012 an international consortium of scientists, clinicians, epidemiologists, and policy makers from public health, industry, medicine, and food regulatory sectors convened in Brussels to begin the process of planning out the global modernization of infectious disease diagnostics, surveillance, transmission, and outbreak investigation through adoption of NGS technologies (Aarestrup et al., 2012).

## The Global Microbial Identifier Consortium

With accumulated evidence that NGS is more powerful than historical molecular subtyping methods, and fast becoming more cost effective, pressures emerged to begin applying WGS for food safety. However, significant gaps remained to complicate widespread adoption of WGS: For one, *how would communication and multijurisdictional sharing of the large-scale WGS information be achieved for successful disease surveillance?* Fortunately the scientific community engaged early with public health, industry, clinicians, and food regulatory representatives to consider the broad needs of the global community. Such proactive, multi sector engagement and collaboration led to the creation of the Global Microbial Identifier (GMI) consortium (Wielinga et al., 2017), which envisions a global, interoperable analytical platform consisting of standardized pathogen genome databases, typing systems, and bioinformatics analysis tools for microbial and infectious disease identification, and diagnostics that will ultimately be made accessible to all nations with basic laboratory infrastructure (Global Microbial Identifier, 2017). Such an interoperable system should benefit not only the *One Health* frontlines at animal/human interfaces, but also food and agrifood industries, regulatory functions, policy makers, etc. Such a universally accessible platform also should benefit broader scientific, R&D and industrial applications.

The GMI vision is as challenging as it is ambitious. To clarify these challenges and develop a way forward, the GMI formed a number of working groups that have been instrumental in advancing genomics and bioinformatics for food safety: WG1 – *Political challenges, outreach and building a global network*; WG2 – *Repository and storage of sequence and associated metadata*; WG3 – *Analytical approaches*; and WG4 – *Methods validation, ring trials and proficiency assurance*. The manifold achievements and progress of these WGs are regularly updated at the GMI web site (Global Microbial Identifier, 2017); in this review we only focus on the activities that relate to open data, bioinformatics and food safety.

### WG1: Political Challenges, Outreach, and Building a Global Network

From the beginning, the GMI WG1 recognized the extreme value of open access and integrated this philosophy as a core principle in its vision. It also appreciated that the adoption of such an approach will require global cooperation and coordination between many different and broad sectors, a large number of which have longstanding policies and laws governing data access and data sharing. In addition, many researchers working in these institutions hold provincial notions about the public health value of the data they possess, and thus are hesitant about rapid release of pathogen genomic data to the public archives. To achieve large-scale, global buy-in to the open data model, the concerns and needs of these stakeholders must be addressed. Thus, the focus of WG1’s activities includes identifying challenges and solutions regarding the varying sensitivities of metadata, intellectual property rights (IPR), and legal implications of open data as they apply to nations, regulatory agencies, and the food industry.

### WG2: Repository and Storage of Sequence and Associated Metadata

GMI WG2 has been dealing with *Storage of sequence and associated contextual metadata*. The group advocates for rapid release of foodborne outbreak pathogen genomic data to the world’s public archives. The group also promotes the standardization of the associated epidemiological, clinical, and laboratory metadata, for the purpose of facilitating data exchange and multijurisdictional approaches to outbreak control (Aarestrup and Koopmans, 2016).

To address concerns regarding the value of rapid release of standardized epidemiological metadata to the public domain versus the potential risk(s) that such information might expose to the institutions and nations contributing data, WG2 addressed the requisite issue of standardization. WG2 worked to enable a minimal common language for rapid release of pathogen genomic data that minimizes the legal risk of public data sharing while retaining the ability to conduct multinational outbreak investigations in real time (Aarestrup and Koopmans, 2016). Its solution exploits the fact that person-sensitive epidemiological data is not always required in order to detect emerging threats and outbreaks—contextual data (e.g., source country, year of isolation, origin, and whether (or not) it derived from an infection) are often sufficient. Consequently, such a minimum set of contextual data was developed (using controlled vocabularies) as the new MDM (or Minimal Data for Matching) reporting standard for data repository submissions of genome-scale pathogen sequence data (GMI meeting report 6). Both the US-hosted National Center for Biotechnology Information (NCBI)’s Short Read Archive (SRA) and the European Molecular Biology Laboratory (EMBL)’s European Nucleotide Archive (ENA) have adopted the GMI’s MDM standard as minimal information fields to be reported for large-scale bacterial genome sequencing projects.

### WG3: Analytical Approaches

GMI WG3 has been dealing with *Analytical approaches*, aiming to define the functional requirements of the major applications (e.g., typing, surveillance, diagnostics) into the global platform, and the analytical systems to be implemented to convert raw pathogen sequence data into actionable knowledge for public health and food regulatory response. WG3 has completed the mapping of the current analytical options and solutions against the needs of GMI end users; the group is currently developing systems for standardizing the comparison of different analytical pipelines. The group also has been active in developing benchmark datasets that can be used to validate the analytical pipelines as well as calibrating them to a common standard such that the results generated can be globally shared, compared, and consistently interpreted.

### WG4: Methods Validation, Ring Trials, and Proficiency Assurance

GMI WG4 has endeavored to survey and promote partner lab consistency in both NGS data generation and data analyses, thereby ensuring that shared NGS data will remain high quality and reliable. WG4 previously established a proficiency testing framework, and has run two full-sized, global proficiency tests focused on assessing the quality of partner lab sequencing of bacterial isolates and of control DNA, and of performing cluster analysis on sets of bacterial genome datasets (Moran-Gilad et al., 2015b; Reinert et al., 2015); PT2016 underway at time of writing). The early trials focused on the foodborne bacterial pathogens *E. coli* and *Salmonella enterica* Serovar Typhimurium; current trials are evaluating the foodborne pathogens *Listeria monocytogenes* and *Campylobacter* spp., and antimicrobial resistant *Klebsiella*. Future WG4 efforts aim to broaden analyses to include viral pathogens.

## Modernizing Food Safety with Genomics, Bioinformatics, and Open Data Access

Several large-scale pilot projects have been implemented that apply NGS and modern bioinformatics analyses to existing foodborne disease surveillance programs. More specifically, these programs are aimed at replacing current subtyping approaches that underpin much of the modern food safety lab operations, with WGS data for real-time molecular surveillance. These modernization efforts represent one of the most crucial transformations in the history of food safety, with benefits and overall impact only starting to be realized. To get a sense of the scale required for this shift, it is important to review the role of molecular subtyping in infectious disease surveillance and control.

At its most basic level, subtyping is used to discriminate strains from the same species and to infer genetic relatedness, linking clinical cases representing a possible outbreak and further linking them to potential sources of infection (Sabat et al., 2013). More often, this goal is challenging to achieve amongst a background of sporadic cases in the absence of clear epidemiological links (Boxrud et al., 2010; Tauxe et al., 2010). Use of standardized laboratory protocols, standardized approaches for analysis and interpretation of data, and a common convention for naming molecular subtypes, collectively have been critical to large-scale deployment of subtyping for routine surveillance. The latter are best achieved in a public or open model, such as in the case of MLST where publicly available databases such as pubMLST (2017) are used by the global community as repositories for shared subtyping data, providing a means for efficient and open data exchange. A different model is sometimes necessary where, due to privacy concerns, restricted networks are required for secure data exchange. An example of this model has been the PulseNet network, which operates as an interconnected virtual laboratory network for the exchange of PFGE data by trusted members (namely laboratories of public health authorities and food safety regulators).

In considering WGS as a replacement for current subtyping methods, it is worth noting that in molecular epidemiology the key assumption is that subtyping data is a proxy for the underlying genomic information from which it is derived. Existing typing methods can thus be viewed as temporary solutions in an era when rapid and inexpensive WGS was not possible; emergence of NGS and adoption of WGS are solving this limitation for public health and food safety investigations. Although WGS data can be analyzed using a traditional phylogenetic framework, the application of NGS in epidemiological surveillance requires approaches for WGS-based subtyping and additionally for relating WGS data to a subtype via a nomenclature scheme. WGS-subtyping facilitates efficient analysis of WGS data and is essential given the exponential increase in available data. A nomenclature is vital to the communication of results to public health or food safety professionals, allowing the monitoring of epidemiological trends and facilitating a rapid response aimed at disease prevention and control.

Of the two main strategies proposed for WGS-based subtyping, the first is based on the analysis of single nucleotide variant [SNV; also called single nucleotide polymorphism (SNP)] and small insertions/deletions (indels) between strains. Although this type of analysis can be performed on draft genome assemblies, several tools have been developed that directly compare raw sequence reads to a related reference genome sequence (Reinert et al., 2015). This process, which is referred to as variant detection by reference mapping, relies on algorithms that align each read to a reference genome and index the variation between them, also assigning confidence levels to each variant position based upon the sequence coverage and level of agreement between reads supporting the SNV (Mielczarek and Szyda, 2016). Reference mapping methodology has been used extensively in studies that have successfully used WGS in outbreak investigations (Harris et al., 2010; Gardy et al., 2011; Grad et al., 2012; Köser et al., 2012; Chewapreecha et al., 2014; Revez et al., 2014; Bekal et al., 2016). Reference mapping also is the approach that has been employed in analyzing *S. enterica* data within the large-scale, international GenomeTrakr project (Allard et al., 2016).

One of the challenges in reference mapping is that it is not always possible to identify an existing high-quality genome sufficiently similar to the genomes under study as a suitable reference genome. Although a closed and manually curated genome is preferable, it is feasible to apply a standard draft genome as the reference, provided that steps are taken to mask (filter out) regions posing problems for unambiguous read mapping (Lynch et al., 2016); these data can be generated on an *ad hoc* basis during the course of an investigation. Another challenge has been the development of nomenclature schemes for SNV reporting in the context of longitudinal pathogen surveillance. Recently, however, researchers at Public Health England have described an approach for systematically deriving pathogen subtype information based on a SNV-address approach (European Centre for Disease Prevention and Control, 2016). Moreover, because the SNV-based approach focuses on the subtlest form of genetic variation, it can be especially useful when investigating isolates exhibiting low levels of sequence variation, such as is expected when comparing outbreak-related isolates and investigating highly clonal or monomorphic populations (Machado et al., 2017).

The second major strategy for WGS-based subtyping is the ‘gene-by-gene’ approach, based on the original MLST concept (Maiden et al., 1998) but extended to the whole-genome level (wgMLST) (Sheppard et al., 2012; Maiden et al., 2013). MLST is based on indexing variation where each locus, a gene or gene fragment, is used as the basic unit of comparison. It has been proposed as a practical framework for developing hierarchical subtyping/nomenclature schemes suitable for studying strain relationships at a range of different resolution levels (Maiden et al., 2013). These include ribosomal MLST (rMLST) (Jolley et al., 2012), which targets 53 ribosomal protein subunit genes suitable for resolving bacterial isolates at all taxonomic levels; and core genome MLST (cgMLST) (Jolley and Maiden, 2010), which targets the genes shared by all or most members of a species (i.e., core genes). Genome-wide approaches to MLST have been applied to *Campylobacter jejuni* (Sheppard et al., 2012) and several other pathogens (Kohl et al., 2014; de Been et al., 2015; Moran-Gilad et al., 2015a; Pightling et al., 2015; Ruppitsch et al., 2015; Chen et al., 2016; Kluytmans-van den Bergh et al., 2016). The approach recently has been validated in a PulseNet International pilot project performing real-time NGS-based typing of *L. monocytogenes* (Jackson et al., 2016). PulseNet International also recently has committed to the wgMLST approach for their routine surveillance of foodborne disease (Carleton and Gerner-Smidt, 2016).

A drawback of cgMLST is that the numbers of genes in the core for any group of strains are dramatically lower than the total number available in a species ‘pan-genome,’ which is comprised of both the core and any accessory genes present in only some strains (Tettelin et al., 2008). It is possible, however, to design *ad hoc* MLST schemes based on the expanded number of genes shared by a smaller subset of genomes, thus providing additional discriminatory power when a low level of genetic variability is expected, as is the case in a rapidly expanding outbreak (Zhang et al., 2015). In addition, it is possible to extend the approach to whole genome MLST (wgMLST) by indexing allelic variation in both core and accessory genes. A hybrid analysis incorporating variation in core, accessory, and regulatory genome regions has recently been presented for the pathogenic *E. coli* lineage ST131 (McNally et al., 2016). Another potential problem with the gene-by-gene approach is that it collapses the diversity at multiple SNV sites located within a locus into a single allelic variant, greatly reducing discriminatory power. Nevertheless, species that are highly recombinogenic (essentially mosaic genomes) will benefit from this type of analytical treatment if the import of multiple SNVs in a single recombination event is a likely occurrence.

To permit stringent use of WGS data as standard public health practice, quality control metrics (such as sequence coverage) and interpretation criteria are needed. Regretfully, such metrics and criteria are still being defined for the field and remain a “moving target.” Additionally they do vary with bacterial species, the time frame of an investigation, and the methodology undertaken for the analyses; hence, no easy “one size fits all” approach exists. Although it remains premature to describe quality metrics and interpretation criteria in specific terms, key factors influencing sequence data generation have been revealed (at least for the mature sequencing technologies) and there are ongoing global efforts to formalize how to generate reliable data and how to robustly interpret the data with confidence. The task is a difficult one since the sequencing parameters, timeframe of analysis, and evolutionary dynamics of the organism all influence the correlation of genomic variation and epidemiological interpretation in a complex way. Owing to the importance of data generation and interpretation in foodborne outbreak investigations, they remain high priorities and will receive considerable attention for the foreseeable future.

## The Proliferation of Bioinformatics Software for Infectious Disease Analyses

The shift in policy amongst food regulatory and public health agencies to rapidly release their pathogen genomic sequence data to the public allowed for the academic research community to join with government scientists, not just in the analysis of foodborne pathogen genomic data, but also in the creation of bioinformatics tools that can store, manage, and analyze the data. Additionally for the first time, genome sequences were being made publicly available for entire populations of pathogens, which spurred innovation in novel types of analyses performed, and in the development of “big data” approaches to efficiently analyze these vast datasets. The number and variety of bioinformatics software developed to analyze microbial data has grown tremendously, and is beyond any kind of comprehensive review. Here we report on some of the most popular and innovative bioinformatics software developed to tackle the analysis of large pathogen genome datasets with a focus on their application in food safety. For more in-depth reviews of the major bioinformatics pipelines used in foodborne disease surveillance, outbreak response, and diagnostics development, we refer the reader to the literature (Lynch et al., 2016; Ronholm et al., 2016).

Some of the first pipelines developed to facilitate analysis of large numbers of pathogen genomes were designed to generate phylogenies from whole genome sequence data. The variation identified among the analyzed sequences is used to infer phylogenetic trees providing supporting evidence for (or against) attributing a given isolate as part of an outbreak under study. In the previous section, we introduced two main approaches to capture this variation: SNV-based methods incorporated into pipelines such as the GenomeTrakr’s CFSAN SNP Pipeline (Davis et al., 2015); and gene-by-gene methods incorporated into whole genome MLST-based pipelines such as BIGSdb (Jolley and Maiden, 2010). A third approach, referred to as alignment-free methods, trades accuracy for speed in inferring the genetic distance between large populations of bacterial genomes and is useful for the rough clustering of thousands of genomes. One of the most notable implementations of this approach is Mash (Ondov et al., 2016), which can efficiently cluster upward of 50,000 draft bacterial genomes on a single CPU in just over a day.

A second major area of development is focused on *in silico* prediction of serotype for foodborne pathogens. These systems promise to drastically reduce cost and effort required to perform conventional antibody-based serotype determinations by instead predicting the serotype via analysis of the pathogen draft genome sequence. One such system, named SISTR (*Salmonella In Silico* Typing Resource) boasts an impressively high serovar predictive accuracy (∼95%) (Yoshida et al., 2016). Additional serotype prediction systems have been built for other pathogens with demonstrated high predictive accuracy such as the SerotypeFinder system for *E. coli* (Joensen et al., 2015). It is expected that such systems will replace much of the conventional serotyping in food regulatory and public health labs.

A third field of active bioinformatics development is focused on the prediction of antimicrobial (antibiotic) resistance from NGS data. Some systems such as ResFinder (Kleinheinz et al., 2014) report the antimicrobial resistance genes they find in whole genome sequence data. ResFinder has high accuracy for finding antimicrobial resistance-associated genes, but cannot discriminate between allelic variants and their associated antibiotic resistant or sensitive phenotype. In contrast, the Comprehensive Antimicrobial Resistance Database (CARD) (Jia et al., 2017) incorporates curated models for each antimicrobial resistance gene and thus, can identify genes associated with antimicrobial resistance and also can predict whether they are resistant or sensitive to a given antibiotic or antibiotic class.

These powerful new bioinformatics tools hold great promise to augment or replace modern food safety lab tests and activities. However, to be used routinely in the front lines of foodborne disease surveillance and outbreak investigation, they need to be implemented in robust, user friendly software systems that shield the end user from the enormous complexity required to store, manage, and analyze vast amounts of data involved in these activities. Several commercial systems are available, such as Ridom SeqSphere+ (Ridom GmbH, Münster, Germany) and BioNumerics (Applied Maths, Sint-Martens-Latem, Belgium). These systems combine proprietary and open source analysis pipelines with sophisticated, easy-to-use interfaces that are familiar and intuitive for use by food safety investigators. One notable alternative is the completely open source Integrated Rapid Infectious Disease Analysis (IRIDA) platform, which provides a web-based end-to-end system for the storage, management, analysis, and sharing of NGS data (IRIDA, 2017). The IRIDA system is built to integrate multiple analytical pipelines in a common data storage and analysis system for genomic epidemiological applications. Other similar, albeit more focused, systems that provide easy-to-use interfaces with modern data analysis and visualization capacity include the Microreact system for phylogeographic analysis of SNV or MLST data (Argimón et al., 2016), the PHYLOViZ system (Ribeiro-Gonçalves et al., 2016; Nascimento et al., 2017) for epidemiological analysis and visualization of sequence (SNV and MLST) data, and GenGIS (Parks et al., 2009, 2013), which allows the overlay and analysis of phylogenetic data and associated metadata on digital maps.

## What is Next? Standard Vocabularies for Genomic Epidemiology and Food Safety

As mentioned above, the GMI:MDM standard (developed by the GMI and adopted by the world’s public data archives) provides an important starting point for sharing the publicly available metadata. Yet much more can be achieved by having standards to describe the multiple layers of information associated with samples from microbial infection or food contamination events. This additional information, which is valuable for interpretation purposes, can range from the sample retrieval site (e.g., host-specific sites or environment) additional laboratory test results (e.g., antibiotic resistance profiles or additional typing methodologies), and possible clinical information (e.g., disease severity). The approaches developed to capture this information range from the definitions of a minimum information “checklist” to record the essential data, to fully-fledged ontologies, which provide a formal description of the entities in a given field of knowledge and the relationships between those entities. While their principal application is to create a machine-readable format that can be easily shared and understood between different databases and software, the process of constructing an ontology by domain experts allows the identification of the key concepts and steps that need to be described and shared. The best-known and most influential ontology in the field of molecular biology is the “Gene Ontology,” which aims to provide a formal and descriptive representation of the biological function of genes (Ashburner et al., 2000). Its impact on biology has been profound: by providing a unifying tool that organizes and standardizes the staggering complexity of life, the Gene Ontology allows for the comprehensive analysis of biological function across all biological domains. Its success represents the impact that may be had by applying ontologies to other complex knowledge domains.

Since founding in 2005 and publication of its first seminal paper defining the Minimum Information about a Genome Sequence specification (MIGS) (Field et al., 2008), the Genomic Standards Consortium (GSC) (Genomic Standards Consortium, 2017) has been highly influential. The need to classify and annotate metagenome data also resulted in a refinement to MIGS to include metagenome metadata resulting in MIGS/MIMS (Garrity et al., 2008). Additional specifications such as Minimum Information about a Marker gene Sequence (MIMARKS) and Minimum Information about any (x) Sequence (MIxS) further refined their original standard (Yilmaz et al., 2011). Early GSC standards focused on sampling (geographic location, type of study) and sequencing information. More recently, the consortium has expanded the scope of their standardization efforts to include the environmental context of the biological identities sampled. This effort led to the development of the Environment Ontology (ENVO) that characterizes the sampling from general environmental sampling to specific body sites (Buttigieg et al., 2013).

However, extra effort to properly annotate sequence data and associated contextual metadata using standardized formats is still needed and additional field-specific information layers need to be directly applied to outbreak and population surveillance. Currently, the leading international effort in creating such a framework is being spearheaded by the Genomic Epidemiology Ontology (GenEpiO) consortium (GenEpiO, 2017). The GenEpiO consortium is tackling different aspects of the contextual metadata in order to facilitate the use of current or expanded ontology to genomic epidemiology investigations in clinical, food and environment surveillance and outbreaks. These range from defining specifications for reportable disease surveillance systems to standardizing food vocabularies, to more specific aspects of biological meaning such as describing antimicrobial resistance mechanisms. The latter requires updating and expansion of the Antimicrobial Resistance Ontology (ARO), developed by the Comprehensive Antibiotic Resistance Database (Jia et al., 2017), a manually curated repository of antimicrobial resistance mechanisms.

The need to annotate existing sequence-based microbial typing data also prompted the development of TypON, the microbial typing ontology (Vaz et al., 2014). This ontology focuses on the specification of sequence-based typing methods such as MLST, MLVA or single locus methodologies (e.g., *spa* typing for *S. aureus*, typing of the Short Variable Region (SVR) of Flagellin B for *Campylobacter* typing (Mellmann et al., 2004). The ontology is especially useful for annotating gene-by-gene methods (Sheppard et al., 2012) such as core or whole genome MLST, facilitating the comparison of existing schemas.

Although the application of NGS has brought great advances to epidemiological research over traditional methodologies for strain characterization, the whole process from the sample processing to sequencing and data analysis is more complex, leading to new challenges: multiple protocols for sample and library preparations are available and each sequencing run can use different versions of sequencing units and consumable reagents; moreover, in terms of data analysis, there are potentially hundreds of different software and respective versions that can be used and need to be tracked. Therefore, to facilitate comparative analyses the entire process from sample to analysis of results should be annotated. This need to capture process led to the creation of the Next Generation Sequencing Ontology (NGSOnto) (NGSOnto ontology, 2017). Using this ontological approach, researchers can maintain a description of the entire lab and data workflow, from sample collection to final results, thereby allowing for assessment of the experimental and bioinformatics pipelines for potential impacts on the resulting data interpretation.

All these efforts for standardization should contribute to a future in which data exchange may seamlessly occur, and truly interoperable resources may be created and shared (Sansone et al., 2012). Standardization and sharing will allow everyone globally to make the most use of the wealth of research and real world data that is being created via NGS technologies.

## The Future is Bright for Bioinformatics, Genomics, Open Access, and Food Safety

The first generation of genomic sequencing methods and analysis pipelines are now in the final stages of translation into routine application in modern food safety labs, and may soon replace many conventional laboratory tests. These advanced genomic and bioinformatics systems have proven their worth in reducing response times to emerging foodborne disease outbreaks, with substantial socioeconomic benefits in terms of improved public health, reduced health care costs, and avoidance of lost productivity due to illness (Scharff et al., 2016). The ongoing global efforts to modernize our food safety systems with genomics and bioinformatics have been impressive, but there remain many challenges and opportunities. Our current analytical capacity still requires the culturing of bacterial isolates, which can take several days. Culture-independent diagnostic testing using metagenomic technologies promises to do away with requisite culturing of isolates, thus shortening our response times even more. Culture independent metagenomics techniques have their own problems, however, such as the large amount of non-target data generated, contamination from environmental sources, and a current inability to distinguish between sequences derived from live or dead microorganisms. The vast and ever-growing size of the pathogen genome databases requires substantial high performance computing resources and novel algorithmic approaches to analyze such large data sets on a useful timescale. Addressing issues of data sharing and data ownership have only just begun. Metadata standardization is making good progress, but will require considerable sustained effort over multiple years to reach maturity. Many of the software pipelines and classification schemas still require extensive validation that will inevitably happen as more data is generated; hence we should anticipate some fluidity for both pipelines and schemas into the future. Substantial effort will be required to increase our capacity to interpret this new type of comprehensive data and to train clinicians and epidemiologists in its use. As mentioned, quality control and quality analysis systems and metrics still need to be developed and standardized. Also, while open data access already has proven beneficial, it likely is not yet feasible for all food safety labs to achieve open data exchange in the immediate short term. However, mounting evidence is emerging for the public health and socioeconomic benefits of open access, plus the availability of bioinformatics tools and computing resources for all; and as these concepts are realized, they will drive a broader policy shift toward openness. Despite this daunting list of challenges that await resolution, implementation of genomics and bioinformatics technologies will occur, and without question will continue to transform our capacity to track and respond to foodborne disease threats.

## Author Contributions

All authors listed, have made equal, direct and intellectual contributions to the work, and approved it for publication.

## Conflict of Interest Statement

The authors declare that the research was conducted in the absence of any commercial or financial relationships that could be construed as a potential conflict of interest.
